# 2-Ethyl-8-methoxy­methyl-4-oxo-4*H*-chromen-7-yl (1*S*,4*R*)-4,7,7-trimethyl-3-oxo-2-oxabicyclo­[2.2.1]heptane-1-carboxyl­ate

**DOI:** 10.1107/S1600536810018921

**Published:** 2010-05-26

**Authors:** Ya Qiu, Ying Chen, Peng Xia

**Affiliations:** a Department of Medicinal Chemistry, School of Pharmacy, Fudan University, Shanghai 201203, People’s Republic of China

## Abstract

The title compound C_23_H_26_O_7_, was prepared by esterification of 2-ethyl-7-hydr­oxy-8-methoxy­methyl-4*H*-chromen-4-one with (*S*)-(−)-camphanic chloride. The two rings of the chromone system are coplanar, making a dihedral angle of 1.99 (19)°, and the camphanoyl unit substituted at 7-*O* retains the original bicyclo­[2.2.1]heptane conformation of the starting reagent.

## Related literature

For background to 3′*R*,4′*R*-Di-*O*-(−)-camphanoyl-2′,2′-di­methyl­dihydro­pyrano[2,3-*f*]chromone (DCP) analogues as potent anti-HIV agents, see: Yu *et al.* (2004 ▶).
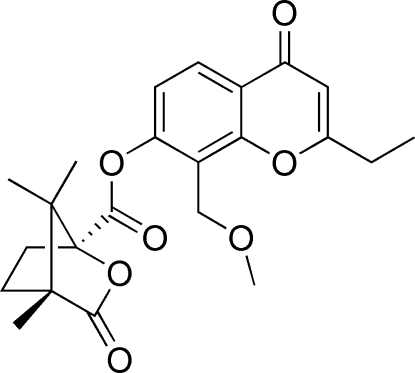


## Experimental

### 

#### Crystal data

C_23_H_26_O_7_
*M* *_r_* = 414.44
Monoclinic, 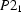

*a* = 7.632 (3) Å
*b* = 14.159 (6) Å
*c* = 10.579 (4) Å
β = 109.240 (5)°
*V* = 1079.3 (8) Å^3^
*Z* = 2
Mo *K*α radiation
μ = 0.09 mm^−1^
*T* = 293 K
0.22 × 0.12 × 0.05 mm

#### Data collection

Bruker SMART APEX CCD area-detector diffractometer
Absorption correction: multi-scan (*SADABS*; Bruker, 2000 ▶) *T*_min_ = 0.980, *T*_max_ = 0.995
4560 measured reflections
2016 independent reflections
1713 reflections with *I* > 2σ(*I*)
*R*_int_ = 0.056

#### Refinement

*R*[*F* ^2^ > 2σ(*F* ^2^)] = 0.046
*wR*(*F* ^2^) = 0.120
*S* = 0.96
2016 reflections
277 parameters
1 restraint
H-atom parameters constrained
Δρ_max_ = 0.22 e Å^−3^
Δρ_min_ = −0.22 e Å^−3^

### 

Data collection: *SMART* (Bruker, 2000 ▶); cell refinement: *SAINT* (Bruker, 2000 ▶); data reduction: *SAINT*; program(s) used to solve structure: *SHELXS97* (Sheldrick, 2008 ▶); program(s) used to refine structure: *SHELXL97* (Sheldrick, 2008 ▶); molecular graphics: *SHELXTL* (Sheldrick, 2008 ▶); software used to prepare material for publication: *SHELXTL*.

## Supplementary Material

Crystal structure: contains datablocks I, global. DOI: 10.1107/S1600536810018921/bh2283sup1.cif

Structure factors: contains datablocks I. DOI: 10.1107/S1600536810018921/bh2283Isup2.hkl

Additional supplementary materials:  crystallographic information; 3D view; checkCIF report

## Figures and Tables

**Fig. 1.**
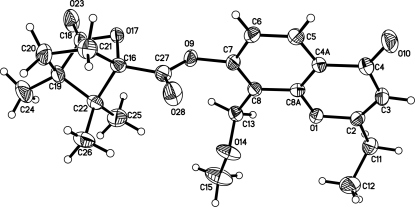
The molecular structure of the title compound showing displacement ellipsoids at the 50% probability level. H atoms are presented as small spheres of arbitrary radius.

## supplementary crystallographic information

### Comment

3'*R*,4'*R*-Di-*O*-(-)-camphanoyl-2',2'-dimethyldihydropyrano[2,3-*f*]chromone(DCP) analogues were potent anti-HIV agents (Yu *et al.* 2004). However, development of DCP analogues as effective anti-AIDS drugs has been hindered by problems of low water solubility and bioavailability. Therefore, we designed and synthesized a series of simplified DCP analogues. As one of the target compounds, the crystal structure of the title compound was reported here.

Fig.1 shows the molecular structure of the title compound, the two rings of chromone are planar and the camphanoyl unit substituted at 7-*O *retains its original bicyclo[2.2.1]heptane  conformation and configuration with the chiralities of C16=S and C19=R in the purchased (S)-(-)-camphanic chloride reagent from Aldrich Company.

### Experimental

The title compound was synthesized by the esterification of 2-ethyl-7-hydroxy-8-(methoxymethyl)-4*H*-chromen-4-one with (*S*)-(-)-camphanic chloride, which was purchased from Aldrich Company. 2-Ethyl-7-hydroxy-8-(methoxymethyl)-4*H*-chromen-4-one (250 mg, 1.07 mmol), (*S*)-(-)-camphanic chloride (255 mg, 1.17 mmol) and DMAP (156 mg, 1.28 mmol) were stirred in CH_2_Cl_2_ (8 ml) for 5 hours at room temperature. The mixture was concentrated and the residue was purified by chromatography on silica gel column with petroleum ether/EtOAc (4:1) as eluent, to afford the pure title compound, which was recrystallized from ethyl acetate to give colorless crystals for the single-crystal X-ray diffraction analysis. Yield: 52.5%.

### Refinement

All H atoms were placed in the idealized positions with C—H = 0.93–0.96 Å and with *U*_iso_(H) = 1.2–1.5 *U*_eq_(C). 1764 Friedel pairs were merged for refinement.

### Crystal data

**Table:**

C_23_H_26_O_7_	*F*(000) = 440
*M**_r_* = 414.44	*D*_x_ = 1.275 Mg m^−^^3^
Monoclinic, *P*2_1_	Mo *K*α radiation, λ = 0.71073 Å
Hall symbol: P 2yb	Cell parameters from 755 reflections
*a* = 7.632 (3) Å	θ = 2.5–24.2°
*b* = 14.159 (6) Å	µ = 0.09 mm^−^^1^
*c* = 10.579 (4) Å	*T* = 293 K
β = 109.240 (5)°	Block, colourless
*V* = 1079.3 (8) Å^3^	0.22 × 0.12 × 0.05 mm
*Z* = 2	

### Data collection

**Table:**

Bruker SMART APEX CCD area-detector diffractometer	2016 independent reflections
Radiation source: fine-focus sealed tube	1713 reflections with *I* > 2σ(*I*)
graphite	*R*_int_ = 0.056
φ and ω scans	θ_max_ = 25.2°, θ_min_ = 2.0°
Absorption correction: multi-scan (*SADABS*; Bruker, 2000)	*h* = −8→9
*T*_min_ = 0.980, *T*_max_ = 0.995	*k* = −16→16
4560 measured reflections	*l* = −10→12

### Refinement

**Table:**

Refinement on *F*^2^	Secondary atom site location: difference Fourier map
Least-squares matrix: full	Hydrogen site location: inferred from neighbouring sites
*R*[*F*^2^ > 2σ(*F*^2^)] = 0.046	H-atom parameters constrained
*wR*(*F*^2^) = 0.120	*w* = 1/[σ^2^(*F*_o_^2^) + (0.0832*P*)^2^] where *P* = (*F*_o_^2^ + 2*F*_c_^2^)/3
*S* = 0.96	(Δ/σ)_max_ < 0.001
2016 reflections	Δρ_max_ = 0.22 e Å^−^^3^
277 parameters	Δρ_min_ = −0.22 e Å^−^^3^
1 restraint	Extinction correction: *SHELXL97* (Sheldrick, 2008), Fc^*^=kFc[1+0.001xFc^2^λ^3^/sin(2θ)]^-1/4^
0 constraints	Extinction coefficient: 0.013 (4)
Primary atom site location: structure-invariant direct methods	

### Fractional atomic coordinates and isotropic or equivalent isotropic displacement parameters (Å^2^)

**Table:**

	*x*	*y*	*z*	*U*_iso_*/*U*_eq_	
O1	−0.1007 (3)	1.16766 (13)	0.3383 (2)	0.0443 (5)	
C2	−0.0842 (5)	1.2628 (2)	0.3643 (3)	0.0460 (8)	
C3	0.0805 (5)	1.3053 (2)	0.4130 (3)	0.0510 (8)	
H3	0.0838	1.3703	0.4260	0.061*	
C4	0.2530 (5)	1.2546 (2)	0.4464 (3)	0.0512 (8)	
C4A	0.2332 (5)	1.1529 (2)	0.4138 (3)	0.0442 (7)	
C5	0.3855 (5)	1.0927 (2)	0.4357 (4)	0.0520 (8)	
H5	0.5048	1.1176	0.4683	0.062*	
C6	0.3629 (5)	0.9984 (2)	0.4103 (3)	0.0499 (8)	
H6	0.4650	0.9587	0.4267	0.060*	
C7	0.1816 (5)	0.9628 (2)	0.3586 (3)	0.0428 (8)	
C8	0.0253 (4)	1.0176 (2)	0.3351 (3)	0.0384 (7)	
C8A	0.0561 (4)	1.1149 (2)	0.3633 (3)	0.0392 (7)	
O9	0.1590 (3)	0.86439 (13)	0.3419 (2)	0.0448 (6)	
O10	0.4071 (4)	1.29131 (18)	0.4995 (3)	0.0788 (9)	
C11	−0.2707 (5)	1.3072 (3)	0.3325 (4)	0.0577 (9)	
H11A	−0.2553	1.3752	0.3397	0.069*	
H11B	−0.3252	1.2873	0.3990	0.069*	
C12	−0.4034 (7)	1.2838 (4)	0.1956 (5)	0.0941 (16)	
H12A	−0.3525	1.3048	0.1288	0.141*	
H12B	−0.5198	1.3149	0.1827	0.141*	
H12C	−0.4225	1.2167	0.1881	0.141*	
C13	−0.1664 (4)	0.9791 (2)	0.2854 (3)	0.0443 (7)	
H13A	−0.1648	0.9127	0.3086	0.053*	
H13B	−0.2439	1.0123	0.3274	0.053*	
O14	−0.2395 (3)	0.9897 (2)	0.1462 (2)	0.0717 (9)	
C15	−0.4332 (6)	0.9722 (5)	0.0981 (5)	0.1000 (19)	
H15A	−0.4557	0.9064	0.1084	0.150*	
H15B	−0.4824	0.9890	0.0051	0.150*	
H15C	−0.4928	1.0093	0.1481	0.150*	
C16	0.1813 (4)	0.7210 (2)	0.2305 (3)	0.0403 (7)	
O17	0.1708 (3)	0.68204 (13)	0.35476 (19)	0.0431 (5)	
C18	0.0979 (5)	0.5937 (2)	0.3243 (3)	0.0497 (8)	
C19	0.0611 (6)	0.5780 (2)	0.1774 (3)	0.0537 (9)	
C20	0.2585 (7)	0.5689 (3)	0.1692 (4)	0.0729 (12)	
H20A	0.2545	0.5498	0.0802	0.087*	
H20B	0.3312	0.5234	0.2338	0.087*	
C21	0.3406 (6)	0.6700 (3)	0.2025 (4)	0.0635 (10)	
H21A	0.4521	0.6700	0.2804	0.076*	
H21B	0.3679	0.6979	0.1272	0.076*	
C22	0.0037 (5)	0.6798 (2)	0.1255 (3)	0.0510 (8)	
O23	0.0780 (5)	0.54276 (18)	0.4081 (3)	0.0817 (10)	
C24	−0.0689 (8)	0.4966 (3)	0.1177 (5)	0.0948 (16)	
H24A	−0.0131	0.4384	0.1579	0.142*	
H24B	−0.0920	0.4941	0.0229	0.142*	
H24C	−0.1840	0.5057	0.1344	0.142*	
C25	−0.1748 (6)	0.7137 (4)	0.1453 (5)	0.0755 (12)	
H25A	−0.2779	0.6786	0.0878	0.113*	
H25B	−0.1921	0.7797	0.1238	0.113*	
H25C	−0.1669	0.7042	0.2369	0.113*	
C26	−0.0086 (8)	0.6956 (3)	−0.0214 (4)	0.0833 (14)	
H26A	−0.1135	0.6619	−0.0794	0.125*	
H26B	0.1028	0.6729	−0.0344	0.125*	
H26C	−0.0228	0.7618	−0.0419	0.125*	
C27	0.1974 (5)	0.8265 (2)	0.2372 (3)	0.0457 (8)	
O28	0.2358 (5)	0.87123 (19)	0.1549 (3)	0.0764 (9)	

### Atomic displacement parameters (Å^2^)

**Table:**

	*U*^11^	*U*^22^	*U*^33^	*U*^12^	*U*^13^	*U*^23^
O1	0.0449 (12)	0.0358 (11)	0.0486 (12)	0.0011 (10)	0.0106 (10)	−0.0002 (9)
C2	0.057 (2)	0.0398 (17)	0.0396 (16)	0.0053 (16)	0.0137 (14)	0.0023 (13)
C3	0.063 (2)	0.0355 (17)	0.053 (2)	−0.0031 (15)	0.0165 (17)	−0.0045 (13)
C4	0.051 (2)	0.0424 (19)	0.054 (2)	−0.0078 (15)	0.0098 (15)	−0.0022 (15)
C4A	0.049 (2)	0.0363 (16)	0.0424 (17)	−0.0044 (14)	0.0083 (14)	−0.0008 (13)
C5	0.0417 (18)	0.0453 (19)	0.059 (2)	−0.0062 (15)	0.0033 (15)	0.0011 (16)
C6	0.0422 (18)	0.0421 (18)	0.0583 (19)	0.0065 (15)	0.0067 (15)	0.0036 (15)
C7	0.053 (2)	0.0324 (16)	0.0409 (16)	−0.0031 (14)	0.0127 (14)	0.0044 (12)
C8	0.0441 (17)	0.0369 (16)	0.0331 (14)	−0.0060 (13)	0.0111 (12)	0.0006 (11)
C8A	0.0431 (17)	0.0388 (17)	0.0331 (15)	0.0015 (14)	0.0091 (12)	0.0032 (12)
O9	0.0593 (15)	0.0297 (11)	0.0472 (12)	−0.0018 (10)	0.0199 (11)	0.0027 (9)
O10	0.0570 (17)	0.0515 (16)	0.114 (2)	−0.0144 (13)	0.0092 (15)	−0.0151 (16)
C11	0.061 (2)	0.049 (2)	0.062 (2)	0.0101 (17)	0.0192 (18)	−0.0024 (16)
C12	0.084 (3)	0.091 (4)	0.082 (3)	0.031 (3)	−0.007 (2)	−0.005 (3)
C13	0.0460 (18)	0.0434 (17)	0.0442 (16)	−0.0052 (14)	0.0158 (14)	−0.0030 (14)
O14	0.0460 (14)	0.123 (3)	0.0396 (12)	−0.0274 (15)	0.0050 (10)	−0.0020 (15)
C15	0.050 (2)	0.169 (6)	0.068 (3)	−0.030 (3)	0.002 (2)	−0.007 (3)
C16	0.0455 (18)	0.0424 (16)	0.0326 (14)	−0.0022 (14)	0.0122 (13)	0.0018 (12)
O17	0.0614 (14)	0.0323 (11)	0.0345 (11)	−0.0037 (9)	0.0144 (10)	−0.0016 (8)
C18	0.072 (2)	0.0333 (16)	0.0446 (18)	−0.0032 (15)	0.0209 (16)	−0.0025 (14)
C19	0.074 (2)	0.0399 (17)	0.0445 (18)	−0.0042 (17)	0.0159 (17)	−0.0093 (14)
C20	0.100 (3)	0.059 (2)	0.064 (2)	0.021 (2)	0.033 (2)	−0.0100 (19)
C21	0.060 (2)	0.073 (3)	0.063 (2)	0.008 (2)	0.0279 (19)	−0.003 (2)
C22	0.058 (2)	0.0506 (19)	0.0356 (16)	−0.0047 (16)	0.0033 (14)	−0.0042 (14)
O23	0.152 (3)	0.0401 (14)	0.0610 (17)	−0.0170 (16)	0.0458 (18)	0.0006 (12)
C24	0.134 (4)	0.056 (3)	0.078 (3)	−0.028 (3)	0.013 (3)	−0.023 (2)
C25	0.051 (2)	0.079 (3)	0.083 (3)	0.002 (2)	0.003 (2)	−0.002 (2)
C26	0.114 (4)	0.081 (3)	0.036 (2)	0.000 (3)	−0.001 (2)	0.0025 (19)
C27	0.051 (2)	0.0419 (17)	0.0427 (18)	−0.0075 (15)	0.0140 (15)	0.0026 (14)
O28	0.122 (2)	0.0554 (16)	0.0639 (16)	−0.0216 (16)	0.0474 (17)	0.0042 (13)

### Geometric parameters (Å, °)

**Table:**

O1—C8A	1.361 (4)	C15—H15B	0.9599
O1—C2	1.372 (4)	C15—H15C	0.9599
C2—C3	1.335 (5)	C16—O17	1.452 (3)
C2—C11	1.489 (5)	C16—C27	1.499 (4)
C3—C4	1.438 (5)	C16—C21	1.525 (5)
C3—H3	0.9300	C16—C22	1.555 (5)
C4—O10	1.238 (4)	O17—C18	1.364 (4)
C4—C4A	1.477 (4)	C18—O23	1.190 (4)
C4A—C8A	1.388 (4)	C18—C19	1.502 (5)
C4A—C5	1.397 (5)	C19—C24	1.517 (5)
C5—C6	1.361 (5)	C19—C20	1.543 (6)
C5—H5	0.9300	C19—C22	1.554 (5)
C6—C7	1.403 (5)	C20—C21	1.556 (7)
C6—H6	0.9300	C20—H20A	0.9700
C7—C8	1.375 (4)	C20—H20B	0.9700
C7—O9	1.408 (4)	C21—H21A	0.9700
C8—C8A	1.412 (4)	C21—H21B	0.9700
C8—C13	1.486 (4)	C22—C25	1.522 (6)
O9—C27	1.346 (4)	C22—C26	1.542 (5)
C11—C12	1.506 (6)	C24—H24A	0.9599
C11—H11A	0.9700	C24—H24B	0.9599
C11—H11B	0.9700	C24—H24C	0.9599
C12—H12A	0.9599	C25—H25A	0.9599
C12—H12B	0.9599	C25—H25B	0.9599
C12—H12C	0.9599	C25—H25C	0.9599
C13—O14	1.401 (4)	C26—H26A	0.9599
C13—H13A	0.9700	C26—H26B	0.9599
C13—H13B	0.9700	C26—H26C	0.9599
O14—C15	1.418 (5)	C27—O28	1.189 (4)
C15—H15A	0.9599		
			
C8A—O1—C2	118.9 (3)	O17—C16—C21	106.4 (3)
C3—C2—O1	122.2 (3)	C27—C16—C21	115.0 (3)
C3—C2—C11	127.2 (3)	O17—C16—C22	102.1 (2)
O1—C2—C11	110.6 (3)	C27—C16—C22	116.5 (3)
C2—C3—C4	122.6 (3)	C21—C16—C22	104.3 (3)
C2—C3—H3	118.7	C18—O17—C16	105.9 (2)
C4—C3—H3	118.7	O23—C18—O17	121.5 (3)
O10—C4—C3	123.9 (3)	O23—C18—C19	130.9 (3)
O10—C4—C4A	121.6 (3)	O17—C18—C19	107.6 (3)
C3—C4—C4A	114.5 (3)	C18—C19—C24	114.6 (3)
C8A—C4A—C5	118.6 (3)	C18—C19—C20	102.6 (3)
C8A—C4A—C4	118.6 (3)	C24—C19—C20	115.5 (4)
C5—C4A—C4	122.7 (3)	C18—C19—C22	99.4 (3)
C6—C5—C4A	121.4 (3)	C24—C19—C22	119.2 (3)
C6—C5—H5	119.3	C20—C19—C22	103.0 (3)
C4A—C5—H5	119.3	C19—C20—C21	103.8 (3)
C5—C6—C7	118.3 (3)	C19—C20—H20A	111.0
C5—C6—H6	120.9	C21—C20—H20A	111.0
C7—C6—H6	120.9	C19—C20—H20B	111.0
C8—C7—C6	123.6 (3)	C21—C20—H20B	111.0
C8—C7—O9	118.2 (3)	H20A—C20—H20B	109.0
C6—C7—O9	117.9 (3)	C16—C21—C20	101.1 (3)
C7—C8—C8A	116.0 (3)	C16—C21—H21A	111.6
C7—C8—C13	123.5 (3)	C20—C21—H21A	111.6
C8A—C8—C13	120.6 (3)	C16—C21—H21B	111.6
O1—C8A—C4A	123.0 (3)	C20—C21—H21B	111.6
O1—C8A—C8	114.9 (3)	H21A—C21—H21B	109.4
C4A—C8A—C8	122.2 (3)	C25—C22—C26	109.5 (3)
C27—O9—C7	116.5 (2)	C25—C22—C19	113.9 (3)
C2—C11—C12	114.4 (3)	C26—C22—C19	114.0 (3)
C2—C11—H11A	108.7	C25—C22—C16	113.1 (3)
C12—C11—H11A	108.7	C26—C22—C16	114.5 (3)
C2—C11—H11B	108.7	C19—C22—C16	91.0 (3)
C12—C11—H11B	108.7	C19—C24—H24A	109.5
H11A—C11—H11B	107.6	C19—C24—H24B	109.5
C11—C12—H12A	109.5	H24A—C24—H24B	109.5
C11—C12—H12B	109.5	C19—C24—H24C	109.5
H12A—C12—H12B	109.5	H24A—C24—H24C	109.5
C11—C12—H12C	109.5	H24B—C24—H24C	109.5
H12A—C12—H12C	109.5	C22—C25—H25A	109.5
H12B—C12—H12C	109.5	C22—C25—H25B	109.5
O14—C13—C8	109.7 (2)	H25A—C25—H25B	109.5
O14—C13—H13A	109.7	C22—C25—H25C	109.5
C8—C13—H13A	109.7	H25A—C25—H25C	109.5
O14—C13—H13B	109.7	H25B—C25—H25C	109.5
C8—C13—H13B	109.7	C22—C26—H26A	109.5
H13A—C13—H13B	108.2	C22—C26—H26B	109.5
C13—O14—C15	111.4 (3)	H26A—C26—H26B	109.5
O14—C15—H15A	109.5	C22—C26—H26C	109.5
O14—C15—H15B	109.5	H26A—C26—H26C	109.5
H15A—C15—H15B	109.5	H26B—C26—H26C	109.5
O14—C15—H15C	109.5	O28—C27—O9	124.3 (3)
H15A—C15—H15C	109.5	O28—C27—C16	122.1 (3)
H15B—C15—H15C	109.5	O9—C27—C16	113.6 (3)
O17—C16—C27	111.3 (2)		
			
C8A—O1—C2—C3	0.1 (4)	C16—O17—C18—C19	−0.5 (4)
C8A—O1—C2—C11	−179.1 (3)	O23—C18—C19—C24	19.0 (7)
O1—C2—C3—C4	−2.1 (5)	O17—C18—C19—C24	−163.2 (4)
C11—C2—C3—C4	176.8 (3)	O23—C18—C19—C20	−107.0 (5)
C2—C3—C4—O10	−175.3 (3)	O17—C18—C19—C20	70.8 (4)
C2—C3—C4—C4A	3.9 (5)	O23—C18—C19—C22	147.3 (5)
O10—C4—C4A—C8A	175.4 (3)	O17—C18—C19—C22	−34.9 (4)
C3—C4—C4A—C8A	−3.8 (4)	C18—C19—C20—C21	−68.6 (3)
O10—C4—C4A—C5	−2.5 (5)	C24—C19—C20—C21	166.0 (4)
C3—C4—C4A—C5	178.3 (3)	C22—C19—C20—C21	34.3 (3)
C8A—C4A—C5—C6	−0.9 (5)	O17—C16—C21—C20	69.0 (3)
C4—C4A—C5—C6	177.0 (4)	C27—C16—C21—C20	−167.3 (3)
C4A—C5—C6—C7	1.2 (5)	C22—C16—C21—C20	−38.4 (4)
C5—C6—C7—C8	−1.6 (5)	C19—C20—C21—C16	2.2 (4)
C5—C6—C7—O9	−175.4 (3)	C18—C19—C22—C25	−64.7 (4)
C6—C7—C8—C8A	1.4 (4)	C24—C19—C22—C25	60.5 (5)
O9—C7—C8—C8A	175.3 (2)	C20—C19—C22—C25	−170.1 (3)
C6—C7—C8—C13	−177.5 (3)	C18—C19—C22—C26	168.6 (4)
O9—C7—C8—C13	−3.7 (4)	C24—C19—C22—C26	−66.1 (5)
C2—O1—C8A—C4A	−0.1 (4)	C20—C19—C22—C26	63.3 (4)
C2—O1—C8A—C8	179.2 (3)	C18—C19—C22—C16	51.2 (3)
C5—C4A—C8A—O1	−179.8 (3)	C24—C19—C22—C16	176.4 (4)
C4—C4A—C8A—O1	2.1 (5)	C20—C19—C22—C16	−54.2 (3)
C5—C4A—C8A—C8	0.9 (5)	O17—C16—C22—C25	63.1 (4)
C4—C4A—C8A—C8	−177.2 (3)	C27—C16—C22—C25	−58.3 (4)
C7—C8—C8A—O1	179.6 (2)	C21—C16—C22—C25	173.7 (3)
C13—C8—C8A—O1	−1.4 (4)	O17—C16—C22—C26	−170.5 (3)
C7—C8—C8A—C4A	−1.1 (4)	C27—C16—C22—C26	68.1 (4)
C13—C8—C8A—C4A	177.9 (3)	C21—C16—C22—C26	−59.9 (4)
C8—C7—O9—C27	110.5 (3)	O17—C16—C22—C19	−53.4 (3)
C6—C7—O9—C27	−75.4 (4)	C27—C16—C22—C19	−174.9 (3)
C3—C2—C11—C12	130.5 (4)	C21—C16—C22—C19	57.2 (3)
O1—C2—C11—C12	−50.4 (4)	C7—O9—C27—O28	−5.3 (5)
C7—C8—C13—O14	−96.3 (4)	C7—O9—C27—C16	176.5 (3)
C8A—C8—C13—O14	84.8 (4)	O17—C16—C27—O28	168.4 (3)
C8—C13—O14—C15	−167.8 (4)	C21—C16—C27—O28	47.3 (5)
C27—C16—O17—C18	160.9 (3)	C22—C16—C27—O28	−75.2 (4)
C21—C16—O17—C18	−73.1 (3)	O17—C16—C27—O9	−13.5 (4)
C22—C16—O17—C18	35.9 (3)	C21—C16—C27—O9	−134.5 (3)
C16—O17—C18—O23	177.6 (4)	C22—C16—C27—O9	102.9 (3)

## References

[bb1] Bruker (2000). *SMART*, *SAINT* and *SADABS* Bruker AXS Inc., Madison, Wisconsin, USA.

[bb2] Sheldrick, G. M. (2008). *Acta Cryst.* A**64**, 112–122. 10.1107/S0108767307043930 18156677

[bb3] Yu, D., Chen, C.-H., Brossi, A. & Lee, K.-H. (2004). *J. Med. Chem.* **47**, 4072–4082 10.1021/jm0400505 15267246

